# Supplementary material for: Diel gene expression improves software prediction of cyanobacterial operons
Source: PeerJ. 2022 Apr 11;10:e13259. doi: 10.7717/peerj.13259 (PMC9009326; doi:10.7717/peerj.13259)
Supplement: Supplemental Information 1 — Classifier names are given as Java class names in the WEKA library. The LMT (Logistic Model Tree) classifier was chosen for this study. [file peerj-10-13259-s001.docx]

| **Classifier** | **Accuracy** |
| --- | --- |
| trees.LMT | 95.28 |
| functions.MultilayerPerceptron | 95.03 |
| functions.Logistic | 92.8 |
| meta.MultiClassClassifier | 92.8 |
| meta.RandomizableFilteredClassifier | 91.89 |
| functions.SimpleLogistic | 91.56 |
| lazy.IBk | 91.31 |
| trees.RandomForest | 91.14 |
| lazy.KStar | 90.89 |
| meta.Bagging | 89.82 |
| meta.RandomCommittee | 89.32 |
| trees.RandomTree | 88.91 |
| functions.SGD | 88.49 |
| meta.MultiClassClassifierUpdateable | 88.49 |
| trees.REPTree | 88.49 |
| trees.J48 | 88.25 |
| rules.JRip | 87.83 |
| meta.RandomSubSpace | 87.67 |
| functions.VotedPerceptron | 87.33 |
| rules.DecisionTable | 87.17 |
| meta.LogitBoost | 86.51 |
| meta.FilteredClassifier | 86.26 |
| meta.IterativeClassifierOptimizer | 86.09 |
| rules.PART | 85.6 |
| meta.AdaBoostM1 | 85.51 |
| functions.SMO | 85.1 |
| bayes.BayesNet | 84.44 |
| trees.HoeffdingTree | 83.53 |
| bayes.NaiveBayesMultinomial | 83.44 |
| bayes.NaiveBayesMultinomialUpdateable | 83.44 |
| bayes.NaiveBayes | 82.7 |
| bayes.NaiveBayesUpdateable | 82.7 |
| meta.AttributeSelectedClassifier | 82.12 |
| lazy.LWL | 81.71 |
| trees.DecisionStump | 81.71 |
| rules.OneR | 81.29 |
| bayes.NaiveBayesMultinomialText | 66.14 |
| functions.SGDText | 66.14 |
| meta.CVParameterSelection | 66.14 |
| meta.MultiScheme | 66.14 |
| meta.Stacking | 66.14 |
| meta.Vote | 66.14 |
| meta.WeightedInstancesHandlerWrapper | 66.14 |
| misc.InputMappedClassifier | 66.14 |
| rules.ZeroR | 66.14 |
